# Parental Perspectives on Antismoking Discussions With Adolescents in Rural African American Households, May 2004-January 2005

**Published:** 2009-03-15

**Authors:** Susan Butler, Michelle Crozier Kegler, Cam Escoffery

**Affiliations:** Department of Behavioral Sciences and Health Education, Rollins School of Public Health, Emory University; Emory University, Atlanta, Georgia; Emory University, Atlanta, Georgia

## Abstract

**Introduction:**

The purpose of this study was to use qualitative interviews to examine antismoking discussions African American parents and adult family members have with adolescent children. This study is one of the first studies to examine the content of family discussions about not smoking among rural African American families from the perspective of parents and extended family members.

**Methods:**

Interview topics included discussions with their children, how their children reacted to those discussions, expected and actual consequences for their children trying a cigarette, and perspectives on how best to keep their children from becoming cigarette smokers. A total of 72 African American households participated in the overall study, and 112 people were interviewed.

**Results:**

Major themes that emerged included discussing the negative health and economic aspects of smoking and the influence of peer pressure. Likely consequences for trying a cigarette included talking to the child about the dangers of smoking and taking away privileges. Making cigarettes less accessible, continued discussions, leading by example, and not smoking around children were suggested as strategies to keep children from smoking.

**Conclusion:**

This study provides insight into antismoking socialization efforts in rural African American families and confirms that African American families are actively engaged in keeping their children from smoking.

## Introduction

Parental smoking status and home smoking rules are associated with smoking initiation in children (1-4). Several studies suggest that adolescents are less likely to smoke if they perceive parental disapproval of smoking and if their parents set and communicate expectations for not smoking, establish no-smoking rules in the home, make clear the consequences of attempting to smoke, and engage in parental monitoring such as knowing where their child is at all times and with whom (5-7). A recent longitudinal examination found that parental expectations protected early adolescents against smoking initiation, even with an increase in the number of friends who smoked (8). Evidence also suggests that parents who smoke can engage in effective antismoking socialization (5,9).

Results from the 2006 National Survey on Drug Use and Health indicate that a smaller proportion of African American youth aged 12 to 17 years than white youth currently smoke (6.5% versus 14.9%) (10). One possible explanation for this difference may be that African American families practice different antismoking socialization strategies than do white families. Compared with white parents, African American parents engaged in more antismoking socialization practices in the home (11). African American parents were more confident about their ability to influence their child's tobacco use, more likely to set ground rules about tobacco use, and more likely to have discussed rules about tobacco use with their children. In contrast, white parents were more likely to feel powerless to prevent their children from smoking.

A multisite qualitative study of diverse youth reported similar racial/ethnic differences (12). African American adolescents described family as a primary source of antismoking messages, regardless of parental smoking status. African American adolescents described strong messages from parents about not smoking and expected strict punishment from their parents for smoking. African American youth were also more concerned that their parents would think less of them if they smoked than were youth from several other racial/ethnic groups (13,14).

Much of the research on antismoking socialization is conducted from the perspective of adolescents. Less is known about antismoking socialization practices from the perspective of parents and other adult family members. The purpose of this study is to describe antismoking socialization strategies of African American families. Our findings are based on qualitative interviews with African American parents and other adult family members living with a child aged 10 to 14 years. Because cigarette smoking is more prevalent in rural communities than in metropolitan communities, this study focused on rural African American families (14). This research can increase our understanding of how African American families may affect smoking initiation among their children and provide insights into possible family-based intervention strategies. We present the expected and actual consequences of adolescent children trying a cigarette, parents' antismoking discussions with their children, how the children reacted to those discussions, parental monitoring behaviors, and perspectives on how best to keep children from becoming cigarette smokers.

## Methods

### Sample and setting

This research was part of a larger study that examined the decision-making process families go through to adopt smoke-free home policies (15). Our research focused on African American households. Study participants were parents of children aged 10 to 14 years and other adult household members in 3 counties in rural southwest Georgia. Participants were recruited through newspaper advertisements and fliers distributed in schools, county social service agencies, and other community organizations. Households with a range of smoking restrictions were recruited: households in which none of the adults smoked, at least 1 adult smoked and another did not, and households in which all adults smoked. A total of 72 African American households participated, and 112 people were interviewed. Primary caregivers were interviewed in 40 of the households, and all adult residents were interviewed in the remaining 32. The study was reviewed and approved by Emory University's institutional review board. Signed informed consent was obtained from all participants, and participants received a $35 gift card as compensation for their time.

### Measures and procedures

The interview guide was designed for the larger research study and is described in more detail elsewhere (15). Closed-ended questions covered demographic characteristics, smoking behaviors, and antismoking socialization strategies, such as parental monitoring and frequency of antismoking discussions. Relevant open-ended questions asked about the content of antismoking discussions with their children, reactions, likely consequences for the children if caught smoking, and suggestions for keeping children from smoking.

Interviews were conducted between May 2004 and January 2005 in participants' homes and typically lasted 60 to 90 minutes. In all cases except 1, interviewers matched the respondents in terms of race/ethnicity and sex. When households contained both male and female respondents, 2 interviewers were used. The interviews were tape-recorded and later transcribed verbatim.

### Data analysis

Research assistants for the project listened to each tape and corrected the transcripts when necessary. A codebook was created to capture major themes for each topic covered in the interviews. Two coders coded each transcript independently, and discrepancies were resolved through consensus. QSR-N6 (Praxis Research, Calgary, Alberta) was used for data storage, retrieval, and analysis (16). Content analysis was performed on coded text to identify themes, and matrices were constructed to help identify patterns by household ban status and respondent smoking status. For the closed-ended items in the interview, the statistical program Epi Info (Centers for Disease Control and Prevention, Atlanta, Georgia) was used for data entry, and SPSS version 13.0 (SPSS Inc, Chicago, Illinois) was used for descriptive analyses.

## Results

### Description of study participants

A total of 58 (51.8%) participants were current smokers. Most of those interviewed (71.4%) were women who were mothers, grandmothers, and aunts (Table 1). A total of 76.8% of the respondents reported an annual household income of less than $25,000, and 22.3% reported having less than a high school education. Forty-three percent of households consisted of a single parent with another adult (Table 2). Almost 40% of households included both adult smokers and nonsmokers. The mean number of children and smokers in the home was 2.3 and 1.1, respectively.

### Antismoking discussions with children

Participants were asked if they had ever had a discussion about cigarettes with their child and, if so, to elaborate on the information discussed and how their child responded. Fifty-seven percent of the participants said they frequently talked to their child about not smoking. The major topics discussed included the negative health and economic consequences of smoking and the influence of peer pressure.

**Negative health consequences of smoking**

When asked about the antismoking topics discussed with their child, most participants indicated that they discussed in detail some of the negative health effects of smoking. Although this topic was a strong theme among both smoking and nonsmoking participants, it was more common among nonsmokers. The conversations on negative health consequences often focused on personal testimonies about tobacco-related sickness, disease, and death of family members.

One father explained, "I use myself as an example, well then, look at your daddy, you know smoking, and I've been smoking ever since I was a teenager. And now I'm just about 50 years old. But look what done happen to me now because I was smoking. I done had a stroke."

**Negative economic consequences of smoking**

Participants attempted to discourage their children from smoking by discussing the economic expense of being a regular cigarette smoker. Many of the participants provided examples of other things that the child could purchase instead of cigarettes. This theme was widespread among both smoking and nonsmoking participants. An example includes a grandfather who is a former smoker, who told his grandson, "I don't see it as adding anything to a person from an economic standpoint. I see it as monies being almost flushed down the drain. I tell him don't pick up the habit because it's a bad habit and that's something you really don't need to waste your money on. You could put money to other uses like buy some food or toys or something, just not on cigarettes 'cause it's bad for your health."

**Influence of peer pressure**

A number of participants discussed the influence of peer pressure and how friends may encourage smoking initiation. A nonsmoking mother said, "I try to point out to him [her son] that a lot of kids pick up cigarettes because they think that it's cool, they think it looks good or they're just with a crowd that does it, and I point out to him, you know, he shouldn't be a follower, just always be a leader, if it's something that he picks up later on in life then it's him, but don't pick it up right now, let him make that choice not just looking at somebody else."

**Children's reaction to antismoking discussions**

After we asked participants about their antismoking discussions with their child, we asked how their child responded to the conversations. The major themes that emerged included the child's reassuring parents and other adult family members that he or she would not smoke and the child's encouraging parents and caregivers to quit smoking themselves.

**Children reassuring their parents**

Of the many children who reassured their parents and other adult family members that they would not smoke, approximately half cited a dislike for cigarettes and a negative attitude toward smoking. A mother who is a former smoker said her daughter told her, "Well, momma, you know, I'm never going to put a cigarette in my mouth [because] I'm not that stupid."

**Children encouraging their parents to quit**

A number of children responded to antismoking discussions by encouraging their parents and other adult family members to quit smoking themselves and by making note of the contradiction of a smoking parent asking her/his child not to smoke. A mother who currently smokes responded, "He has asked questions about cigarettes and why do I smoke cigarettes and where do they come from, and I tell him cigarettes are bad and they can cause cancer, and he tells me all the time that he don't want me smoking because I could have cancer and he could lose me."

### Children's expectations of the consequences of trying a cigarette

When asked if they believed their child had tried a cigarette, most participants among all types of households, including those with smokers, responded no. When participants were asked what their child expected would happen if he or she was caught smoking, most participants said their child expected the parent to be angry, to talk about the dangers of smoking, to deny privileges, or to spank the child.

**Expecting the parent to become angry**

If participants found out that their child had tried smoking a cigarette, many thought that their child would expect them to become angry and upset. An aunt who is a current smoker described what her niece would expect, "She'd know I get mad and that's about it. She'd know I get mad with her because I done talked to her and asked her not to."

**Talking to child about the dangers of smoking**

Many participants thought their child expected to be talked to about the dangers of smoking. A father who currently smokes explained what his son would expect, "I would just talk to him, sit him down and let him know it's not good for you and quit while you can."

**Taking away privileges and activities**

Another common expectation consisted of being denied permission to engage in favorite activities. A nonsmoking mother described, "If I caught him smoking, he knows he'll be in a lot of trouble. And be more talk, probably punishment, things taken away from him that he loves, until he gets the picture that it's a no-no in this household."

**Receiving a spanking**

A number of participants mentioned that their children expected a spanking if they were caught smoking. The comments related to this response were short and to the point. For example, an older sister who is a nonsmoker thought her younger sister would expect "to get a whipping, get fussed at, and everything."

### Actual reactions if child tried smoking

Participants were asked what they would actually do if they found out their child had tried smoking. Two familiar themes emerged, including talking to their child about the dangers of smoking and taking away privileges and activities. Even though these 2 themes were mentioned as an expected response by the child, when mentioned as an actual response, participants were more specific about what they would do.

### Preventing children from trying cigarettes and from becoming smokers

Parents and adult family members were asked what works best to keep their child from trying a cigarette and becoming a smoker. Major themes that emerged included continued talking about the dangers of smoking, leading by example, and if participants were current smokers, quitting smoking or at least not smoking around the children. Some study participants mentioned that they do not allow cigarettes in the home and some smokers mentioned that they do not leave cigarettes where their children can find them. In addition, quantitative data (Table 1) showed a high level of parental monitoring among participants who smoke. Most participants said they knew where their child was at all times (mean [SD] = 4.6 [.81]) and with whom (mean [SD] = 4.5 [.82].

**Continue to talk about the dangers of smoking**

Most of the strong antismoking discussions came from nonsmokers and former smokers. A nonsmoking aunt suggested, "They can talk to them about it, and let them know the dangers of some cigarettes, like secondhand smoking, and stuff like that, is dangerous for them and, you know, causing health problems and all of that."

**Lead by example**

Most of the strong antismoking discussions related to leading by example also came from nonsmokers. A nonsmoking father related a story when he responded, "But I think the best thing we can do is lead by example as a father or mother. I know of friends who say to me, my dad told me not to smoke, but he was standing there smoking a cigarette so I didn't listen to what he said. I watched him." Another way to lead by example is to quit smoking. A stepfather who currently smokes suggested, "Well, mainly me not smoking around her, if I could possibly quit, that would be the biggest help of all."

**If you smoke, don't smoke around the children**

Many participants who currently smoke believed that children whose parents smoke are more likely to smoke themselves. They suggested that parents should not smoke around their children. A father who currently smokes explained how he became a smoker by saying, "I started by watching my parents smoke and go and get cigarettes and playing with them and start smoking and that's how I became [a smoker], so the best thing is do not smoke around your kids and don't be no smoking yourself."

## Conclusion

This study provided a rare perspective on the content of antismoking discussions with rural African American adolescents and how families attempt to prevent them from smoking. To our knowledge, this is the first study in a rural setting to examine African American family members' discussions with their children about not smoking. Our findings are consistent with those from prior research showing that African American parents actively engage in behaviors to prevent tobacco use by setting ground rules and having discussions about smoking (11). Results also support other research findings from the perspective of African American adolescents who report that their parents discuss the effects of cigarette smoking with them and that parents threaten harsh consequences if their children smoke (12,13). Major antismoking messages included personalized stories about the negative health effects of smoking, the economic consequences of smoking, and the influence of peer pressure to smoke.

The study participants also offered antismoking socialization strategies that they perceive to be effective in preventing smoking initiation. To prevent their children from trying cigarettes and becoming regular smokers, study participants mentioned continuing to talk to their children about the dangers of smoking, leading by example, and not smoking around the children. Monitoring, a key antismoking socialization strategy, was also cited as a parenting behavior to prevent substance abuse, cigarette smoking, and other risky behaviors among adolescents (1,6,17,18). Most parents and other adult family members reported high levels of monitoring or knowing where their children were at all times.

This study has a number of limitations. Participants self-selected to be involved in the study and may differ from members of other rural African American families. The study was also conducted in rural counties near the first community in the state to pass a smoke-free policy for public places, including restaurants. Members of the community may have been well-versed in antismoking messages and topics. Because of the study setting and political climate, whether the results can be transferred to other African American communities, both rural and urban, is unknown. Finally, participants may have provided socially acceptable responses.

The findings of this study provide insight into the content of parental antismoking discussions and protective behaviors that may inform family-based interventions to prevent smoking among African American adolescents. Although some family-based antismoking interventions have been developed (19-22), they have had mixed results (19,20). Future research should test the effectiveness of the various messages identified in this study in preventing early adolescent smoking. For example, telling personalized stories about the negative consequences of smoking should be compared with encouraging young people to avoid peer pressure. Family-based interventions could also incorporate messages from important family members in addition to parents, given the findings that a mix of family members (such as grandmothers and aunts) provides antismoking messages within African American households.

## Figures and Tables

**Table 1:** Antismoking Characteristics and Demographics of Selected African American Households, Rural Southwest Georgia, May 2004-January 2005

Participants	Total N = 112	Smokers n = 58	Nonsmokers n = 54
**Sex, n (%)**			
Female	80 (71.4)	38 (47.5)	42 (52.5)
Male	32 (28.6)	20 (62.5)	12 (37.5)
**Household income, n (%)**			
<$25,000	86 (76.8)	52 (60.5)	34 (39.5)
$25,000-$49,000	21 (18.8)	6 (28.6)	15 (71.4)
≥$50,000	5 (4.4)	0 (0.0)	5 (100.0)
**Education, n (%)**			
Less than high school	25 (22.3)	15 (60.0)	10 (40.0)
High school graduate/GED	42 (37.5)	26 (61.9)	16 (38.1)
Some college/college degree	45 (40.2)	17 (37.8)	28 (62.2)
**Antismoking socialization^a^, mean (SD) **			
Talk about not smoking	NC	3.5 (0.75)	3.1 (0.95)^b^
Know where child is	NC	4.6 (0.81)	4.8 (0.52)
Know who child is with	NC	4.5 (0.82)	4.6 (0.74)

Abbreviation: GED, general educational development; NC, not calculated.

a Based on a scale from 1 to 5, with 1 = never and 5 = almost always.

b The difference was significant by a 2-tailed test for paired samples (*t*_100_ = 2.06, *P* = .04).

**Table 2:** Characteristics of Selected African American Households Participating in Study of Antismoking Discussions, Rural Southwest Georgia, May 2004-January 2005

Household characteristics	Total (n = 72)
Both parents	20 (27.8)
Single parent	21 (29.2)
Single parent and other adult	31 (43.0)
**Smoking status, n (%)**	
All adult smokers	28 (38.9)
Mixed smokers/nonsmokers	29 (40.3)
No adult smokers	15 (20.8)
**Smoking rules, n (%)**	
Complete ban	24 (33.3)
Partial ban	43 (59.7)
No ban	5 (7.0)
**Size, mean (SD)**	
No. of adults	1.9 (.6)
No. of children	2.3 (1.1)
No. of smokers	1.1 (.9)
